# Is Ohio’s high school CPR requirement reaching students? A survey of central Ohio high school students

**DOI:** 10.1016/j.resplu.2026.101527

**Published:** 2026-10-08

**Authors:** Tharun H. Nambi, Parth N. Shah, Rolla F. Abu-Arja

**Affiliations:** a Olentangy Orange High School, 2840 E. Orange Rd., Lewis Center, OH 43035, USA; b The Ohio State University Wexner Medical Center, 410 W. 10th Ave., Columbus, OH 43210, USA; c Columbus State Community College, 550 E. Spring St., Columbus, OH 43215, USA; d Department of Pediatrics, Nationwide Children’s Hospital, 700 Children’s Dr., Columbus, OH 43205, USA; e Department of Pediatrics, The Ohio State University College of Medicine, Columbus, OH 43210, USA

To the Editor,

Bystander cardiopulmonary resuscitation (CPR) is provided to only about 40% of the more than 350,000 people in the United States with an out-of-hospital cardiac arrest each year.^1^ Most states require CPR instruction in high school,^2^ with Ohio mandating it since 2017.^3^ Whether that instruction reaches students effectively is unknown.

We surveyed grades 9–12 students at central Ohio high schools using an anonymous online questionnaire distributed between February-April 2026.^4^ The study was approved by the Olentangy District Internal Institutional Review Board. Students were recruited through flyers and teacher announcements. Respondents reported prior CPR-training, its source and recency, ability to call for help and begin chest compressions, perceived importance of CPR (5-point Likert scale), and whether CPR-training should be mandated in school. Optional open-ended items queried training barriers and factors that increased preparedness to perform CPR.

Of 387 responses, 379 were analyzed after excluding 8 that failed an attention check (Table 1). Most respondents were in grade 11 (57.8%) and attended public schools (90.2%). Prior CPR-training was reported by 186 (49.1%) respondents, although only 66 (17.4%) reported school-based training. Prior training did not vary by grade (*χ*^2^ = 0.50, *p* = 0.92). Online courses were the most common training source (48.9%), whereas only 11.3% reported school as their sole source. Most trained respondents (86%) trained >1 year earlier.

**Table 1:** Respondent characteristics, CPR training history, preparedness/attitudes, bivariate and multivariable analysis of factors associated with support for mandatory CPR training.

**Variable**	***N* (%)**
**Respondent characteristics (*n* = 379)**	
Grade 9	17 (4.5%)
Grade 10	51 (13.5%)
Grade 11	219 (57.8%)
Grade 12	92 (24.3%)
Public school	342 (90.2%)
Private school	37 (9.8%)

**CPR training history among trained respondents (*n* = 186)**	
Training source*	
Online video/course	91 (48.9%)
Community class	81 (43.5%)
Workplace/extracurricular training	68 (36.6%)
School-based training	66 (35.5%)
School as sole source	21 (11.3%)
Training recency (*n* = 185)**	
Within the past year	26 (14.1%)
1–2 years ago	108 (58.4%)
More than 2 years ago	51(27.6%)
**Training duration**	
Less than 30 min	19 (10.2%)
30 min–1 h	42 (22.6%)
1–2 h	58 (31.2%)
More than 2 h	67 (36%)

**Preparedness and attitudes amongst trained respondents (*n* = 186)**	
**At least able to initiate CPR and call for help**	
Yes	70 (37.6%)
Somewhat	99 (53.2%)
Not sure	17 (9.1%)
**Biggest worry when performing CPR**	
Doing something wrong	68 (36.6%)
Hurting the person	54 (29%)
Panic or fear	28 (15.1%)
Being judged by others	20 (10.8%)
Not remembering the steps	16 (8.6%)
**Bivariate analyses of CPR familiarity, confidence and attitudes**	
Prior training × mandatory training support	*χ*^2^ = 11.52, *p* < 0.001
Grade × training status	*χ*^2^ = 0.50, *p* = 0.92
Familiarity × confidence	*r* = 0.005, *p* = 0.951
Familiarity by grade	*F* = 1.62, *p* = 0.187
Familiarity by training recency	*F* = 1.30, *p* = 0.277
Confidence by training recency	*F* = 5.26, *p* = 0.006
Confidence by training duration	*F* = 0.72, *p* = 0.543
Perceived importance by grade	*F* = 1.172, *p* = 0.320
Perceived importance by trained status	*t* = −1.446, *p* = 0.149
**Multivariable logistic regression for support for mandatory high school CPR training (*n* = 379)**	
Prior CPR training (vs none)	OR 2.54 (1.48–4.37), *p* = 0.001
Grade 9 vs Grade 11	OR 0.43 (0.14–1.34), *p* = 0.144
Grade 10 vs Grade 11	OR 0.40 (0.20–0.81), *p* = 0.010
Grade 12 vs Grade 11	OR 0.69 (0.37–1.30), *p* = 0.252
Private vs public school	OR 2.47 (0.83–7.40), *p* = 0.106
Perceived importance of CPR	OR 0.90 (0.69–1.18), *p* = 0.450

* Training source was a select-all-that-apply item; percentages may exceed 100%. CPR = cardiopulmonary resuscitation.

** Degrees of freedom for training recency comparisons are based on 185 valid responses; one respondent selected two recency options and was excluded from that comparison *χ*^2^: Chi-square testing, *r*: Pearson correlation *F*: One-way ANOVA, *t*: Independent samples *t*-test, OR = odds ratio.

Among trained respondents, mean CPR familiarity was 3.50 [standard deviation (SD) 0.87] and confidence 3.10 (SD 0.82). Overall, 91% of trained respondents reported being at least somewhat able to call for help and initiate CPR, including 38% answering an unqualified yes. Common concerns included doing something wrong (36.6%), causing harm (29.0%), and panic/fear (15.1%). Confidence declined with time since training (3.35 < 1-year, 3.19 at 1–2 years, 2.80 > 2 years; *p* = 0.006) but not by grade or duration; familiarity did not differ by recency, grade, or duration Perceived importance of CPR was high across all respondents (mean 3.94, SD 1.05) irrespective of training status. Overall, 79.7% supported mandatory high school CPR-training, with greater support among trained than untrained students (87.1% vs. 72.5%). Prior CPR-training was independently associated with support for mandatory training (adjusted OR 2.54, 95% CI 1.48–4.37, *p* = 0.001). Among respondents reporting barriers (*n* = 93), lack of availability at school (24.7%), lack of awareness (18.3%), low motivation (16.1%), and fear (10.8%) were most common. Among those describing preparedness (*n* = 86), extracurricular or job training (25.6%), school curriculum (20.9%), and formal courses (16.3%) were cited most often.

Limitations include self-report, an unvalidated instrument, a single-region convenience sample, and no objective skill measures. Our findings concur with prior studies showing high student interest in CPR training but limited confidence and skill retention post-training.^5^, ^6^, ^7^, ^8^, ^9^

Nearly a decade after Ohio’s state-wide mandate, fewer than 1 in 5 students surveyed reported school-based CPR-training and only 38% of trained respondents said they knew how to begin CPR. Improving implementation, visibility, and reinforcement of existing CPR-training requirements, therefore may be more impactful than expanding mandates alone.

## Declaration of generative AI and AI-assisted technologies in the manuscript preparation process

During the preparation of this work the authors used Claude (Anthropic, PBC), including Claude Opus 5 and Claude Opus 5.5, in order to assist with language editing. After using this tool, the authors reviewed and edited the content as needed and take full responsibility for the content of the published article.

## CRediT authorship contribution statement

**Tharun H. Nambi:** Writing – original draft, Validation, Methodology, Investigation, Formal analysis, Data curation, Conceptualization. **Parth N. Shah:** Writing – review & editing, Investigation. **Rolla F. Abu-Arja:** Writing – review & editing.

## Funding

This research project was funded by the first author.

## Declaration of competing interest

The authors declare that they have no known competing financial interests or personal relationships that could have appeared to influence the work reported in this paper.
